# Assessment of the potential impact of a reminder system on the reduction of diagnostic errors: a quasi-experimental study

**DOI:** 10.1186/1472-6947-6-22

**Published:** 2006-04-28

**Authors:** Padmanabhan Ramnarayan, Graham C Roberts, Michael Coren, Vasantha Nanduri, Amanda Tomlinson, Paul M Taylor, Jeremy C Wyatt, Joseph F Britto

**Affiliations:** 1 Children's Acute Transport Service (CATS), 44B Bedford Row, London, WC1H 4LL, UK; 2 Department of Paediatric Allergy and Respiratory Medicine, Southampton University Hospital Trust, Tremona Road, Southampton, SO16 6YD, UK; 3 Department of Paediatrics, St Mary's Hospital, Paddington, London, W2 1NY, UK; 4 Department of Paediatrics, Watford General Hospital, Vicarage Road, Watford, WD18 0HB, UK; 5 Isabel Healthcare Ltd, Po Box 244, Haslemere, Surrey, GU27 1WU, UK; 6 Centre for Health Informatics and Multiprofessional Education (CHIME), Archway Campus, Highgate Hill, London, N19 5LW, UK; 7 Health Informatics Centre, The Mackenzie Building, University of Dundee, Dundee, DD2 4BF, UK

## Abstract

**Background:**

Computerized decision support systems (DSS) have mainly focused on improving clinicians' diagnostic accuracy in unusual and challenging cases. However, since diagnostic omission errors may predominantly result from incomplete workup in routine clinical practice, the provision of appropriate patient- and context-specific reminders may result in greater impact on patient safety. In this experimental study, a mix of easy and difficult simulated cases were used to assess the impact of a novel diagnostic reminder system (ISABEL) on the quality of clinical decisions made by various grades of clinicians during acute assessment.

**Methods:**

Subjects of different grades (consultants, registrars, senior house officers and medical students), assessed a balanced set of 24 simulated cases on a trial website. Subjects recorded their clinical decisions for the cases (differential diagnosis, test-ordering and treatment), before and after system consultation. A panel of two pediatric consultants independently provided gold standard responses for each case, against which subjects' quality of decisions was measured. The primary outcome measure was change in the count of diagnostic errors of omission (DEO). A more sensitive assessment of the system's impact was achieved using specific quality scores; additional consultation time resulting from DSS use was also calculated.

**Results:**

76 subjects (18 consultants, 24 registrars, 19 senior house officers and 15 students) completed a total of 751 case episodes. The mean count of DEO fell from 5.5 to 5.0 across all subjects (repeated measures ANOVA, p < 0.001); no significant interaction was seen with subject grade. Mean diagnostic quality score increased after system consultation (0.044; 95% confidence interval 0.032, 0.054). ISABEL reminded subjects to consider at least one clinically important diagnosis in 1 in 8 case episodes, and prompted them to order an important test in 1 in 10 case episodes. Median extra time taken for DSS consultation was 1 min (IQR: 30 sec to 2 min).

**Conclusion:**

The provision of patient- and context-specific reminders has the potential to reduce diagnostic omissions across all subject grades for a range of cases. This study suggests a promising role for the use of future reminder-based DSS in the reduction of diagnostic error.

## Background

A recent Institute of Medicine report has brought the problem of medical error under intense scrutiny[1]. While the use of computerized prescription software has been shown to substantially reduce the incidence of medication-related error [2,3], few solutions have demonstrated a similar impact on diagnostic error. Diagnostic errors impose a significant burden on modern healthcare: they account for a large proportion of medical adverse events in general [4-6], and form the second leading cause for malpractice suits against hospitals [7]. In particular, diagnostic errors of omission (DEO) during acute medical assessment, resulting from cognitive biases such as 'premature closure' and 'confirmation bias', lead to incomplete diagnostic workup and 'missed diagnoses' [8]. This is especially relevant in settings such as family practice [9], as well as hospital areas such as the emergency room and critical care [11,12], 20% of patients discharged from emergency rooms raised concerns in a recent survey that their clinical assessment had been complicated by diagnostic error [12]. The use of clinical decision-support systems (DSS) has been one of many strategies proposed for the reduction of diagnostic errors in practice [13]. Consequently, a number of DSS have been developed over the past few years to assist clinicians during the process of medical diagnosis [14-16].

Even though studies of several diagnostic DSS have demonstrated improved physician performance in simulated (and rarely real) patient encounters [17,18], two specific characteristics may have contributed to their infrequent use in routine practice: intended purpose and design. Many general diagnostic DSS were built as 'expert systems' to solve diagnostic conundrums and provide the correct diagnosis during a 'clinical dead-end' [19]. Since true diagnostic dilemmas are rare in practice [20], and the initiative for DSS use had to originate from the physician, diagnostic advice was not sought routinely, particularly since clinicians prefer to store the patterns needed to solve medical problems in their heads [21]. There is, however, evidence that clinicians frequently underestimate their need for diagnostic assistance, and that the perception of diagnostic difficulty does not correlate with their clinical performance [22]. In addition, due to the demands of the information era [23]. diagnostic errors may not be restricted to cases perceived as being difficult, and might occur even when dealing with common problems in a stressful environment under time pressure [24]. Further, most 'expert systems' utilized a design in which clinical data entry was achieved through a controlled vocabulary specific to each DSS. This process frequently took > 15 minutes, contributing to infrequent use in a busy clinical environment [25]. These 'expert systems' also provided between 20 and 30 diagnostic possibilities [26], with detailed explanations, leading to a lengthy DSS consultation process.

In order to significantly affect the occurrence of diagnostic error, it seems reasonable to conclude that DSS advice must therefore be readily available, and sought, during most clinical encounters, even if the perceived need for diagnostic assistance is minor. Ideally, real-time advice for diagnosis can be actively provided by integrating a diagnostic DSS into an existing electronic medical record (EMR), as has been attempted in the past [27,28]. However, the limited uptake of EMRs capable of recording sufficient narrative clinical detail currently in clinical practice indicates that a stand-alone system may prove much more practical in the medium term [29]. The key characteristic of a successful system would be the ability to deliver reliable diagnostic reminders rapidly following a brief data entry process in most clinical situations. ISABEL (ISABEL Healthcare, UK) is a novel Web-based pediatric diagnostic reminder system that suggests important diagnoses during clinical assessment [30,31]. The development of the system and its underlying structure have been described in detail previously [32,33]. The main hypotheses underlying the development of ISABEL were that the provision of diagnostic reminders generated following a brief data entry session in free text would promote user uptake, and lead to improvement in the quality of diagnostic decision making in acute medical settings. The reminders provided (a set of 10 in the first instance) aimed to remind clinicians of important diagnoses that they might have missed in the workup. Data entry is by means of natural language descriptions of the patient's clinical features, including any combination of symptoms, signs and test results. The system's knowledge base consists of natural language text descriptions of > 5000 diseases, in contrast to most 'expert systems' that use complex disease databases [34-36]. The advantages and trade-offs of these differences in system design have been discussed in detail elsewhere [37]. In summary, although the ability to rapidly enter patient features in natural language to derive a short-list of diagnostic suggestions may allow frequent use by clinicians during most patient encounters, variability resulting from the use of natural language for data entry, and the absence of probability ranking, may compromise the accuracy and usefulness of the diagnostic suggestions.

The overall evaluation of the ISABEL system was planned in systematic fashion in a series of consecutive studies [38].

a) An initial clinical performance evaluation: This would evaluate the feasibility of providing relevant diagnostic suggestions for a range of cases when data is entered in natural language. System accuracy, speed and relevance of suggestions were studied.

b) An assessment of the impact of the system in a quasi-experimental setting: This would examine the effects of diagnostic decision support on subjects using simulated cases.

c) An assessment of the impact of the system in a real life setting: This would examine the effects of diagnostic advice on clinicians in real patients in their natural environment.

In the initial performance evaluation, the ISABEL system formed the unit of intervention, and the quality of its diagnostic suggestions was validated against data drawn from 99 hypothetical cases and 100 real patients. Key findings from cases were entered into the system in free text by one of the developers. The system included the final diagnosis in 95% of the cases [39]. This design was similar to early evaluations of a number of other individual diagnostic DSS [40-42], as well as a large study assessing the performance characteristics of four expert diagnostic systems [43]. Since this step studied ISABEL in isolation, and did not include users uninvolved in the development of the system, it was vital to examine DSS *impact *on decision making by demonstrating in the subsequent step that the clinician-DSS combination functioned better than either the clinician or the system working in isolation [44,45]. Evaluation of impact is especially relevant to ISABEL: despite good system performance when tested in isolation, clinicians may not benefit from its advice either due to variability associated with user data entry leading to poor results, or the inability to distinguish between diagnostic suggestions due to the lack of ranking [46]. A previous evaluation of Quick Medical Reference (QMR) assessed a group of clinicians working a set of difficult cases, and suggested that the extent of benefit gained by different users varied with their level of experience [47].

In this study, we aimed to perform an impact evaluation of ISABEL in a quasi-experimental setting in order to quantify the effects of diagnostic advice on the quality of clinical decisions made by various grades of clinicians during acute assessment, using a mix of easy and difficult simulated cases drawn from all pediatric sub-specialties. Study design was based on an earlier evaluation of the impact of ILIAD and QMR on diagnostic reasoning in a simulated environment [48]. Our key outcome measure focused on appropriateness of decisions during diagnostic workup rather than accuracy in identifying the correct diagnosis. The validity of textual case simulations has previously been demonstrated in medical education exercises [49], and during the assessment of mock clinical decision making [50,51].

## Methods

The simulated field study involved recording subjects' clinical decisions regarding diagnoses, test-ordering and treatment for a set of simulated cases, both before and immediately after DSS consultation. The impact of diagnostic reminders was determined by measuring changes in the quality of decisions made by subjects. In this study, the quality of ISABEL's diagnostic suggestion list *per se *was not examined. The study was coordinated at Imperial College School of Medicine, St Mary's Hospital, London, UK between February and August 2002. The study was approved by the Local Research Ethics Committee.

### Subjects

A convenience sample consisting of pediatricians of different grades (senior house officers [interns], registrars [residents] and consultants [attending physicians] from different geographical locations across the UK), and final year medical students, was enrolled for the study. All students were drawn from one medical school (Imperial College School of Medicine, London, UK). Clinicians were recruited by invitation from the ISABEL registered user database which consisted of a mixture of regular users as well as pediatricians who had never used the system after registration. After a short explanation of the study procedure, all subjects who consented for the study were included within the sample.

### Cases

Cases were drawn from a pool of 72 textual case simulations, constructed by one investigator, based on case histories of real children presenting to emergency departments (data collected during earlier evaluation). Each case was limited to between 150 and 200 words, and only described the initial presenting symptoms, clinical signs and basic laboratory test results in separate sections. Since the clinical data were collected from pediatric emergency rooms, the amount of clinical information available at assessment was limited but typical for this setting. Ample negative features were included in order to prevent the reader from picking up positive cues from the text. These cases were then classified into one of 12 different pediatric sub-specialties (e.g. cardiology, respiratory) and to one of 3 case difficulty levels within each specialty (1-unusual, 2-not unusual, and 3-common clinical presentation, with reference to UK general hospital pediatric practice) by the author. This allocation process was duplicated by a pediatric consultant working independently. Both investigators assigned 57 cases to the same sub-specialty and 42 cases to both the same sub-specialty and the same level of difficulty (raw agreement 0.79 and 0.58 respectively). From the 42 cases in which both investigators agreed regarding the allocation of both specialty and level of difficulty, 24 cases were drawn such that a pair of cases per sub-specialty representing two different levels of difficulty (level 1 & 2, 1 & 3 or 2 & 3) was chosen for the final case mix. This process ensured a balanced set of cases representing all sub-specialties and comprising easy as well as difficult cases.

### Data collection website

A customized, password protected version of ISABEL was used to collect data during the study. This differed from the main website in that it automatically displayed the study cases to each subject in sequence, assigned each case episode a unique study number, and recorded time data in addition to displaying ten diagnostic suggestions. Three separate text boxes were provided to record subjects' clinical decisions (diagnoses, tests and treatment) pre- and post-DSS consultation. The use of the customized trial website ensured that subjects proceeded from one step to the next without being able to skip steps or revise clinical decisions already submitted.

### Training

Training was intended only to familiarize subjects with the trial website. During training, all subjects were assigned unique log-in and passwords, and one sample case as practice material. Practice sessions involving medical students were supervised by one investigator in group sessions of 2–3 subjects each. Pediatricians (being from geographically disparate locations) were not supervised during training, but received detailed instructions regarding the use of the trial website by email. Context-specific help was provided at each step on the website for assistance during the practice session. All subjects completed their assigned practice case, and were recruited for the study.

### Study procedure

**Figure 1:**
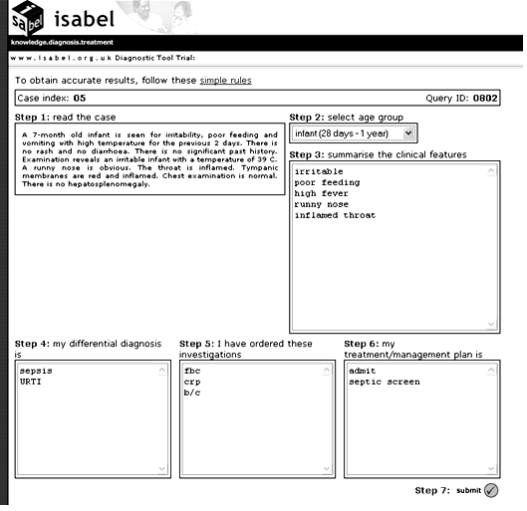
**Screenshot of ISABEL simulated study procedure – step 1**. This figure shows how one subject was presented with the text of a case simulation, how he could search ISABEL by using summary clinical features, and record his clinical decisions prior to viewing ISABEL's results. For this case, clinically important diagnoses provided by the expert panel are: nasopharyngitis (OR viral upper respiratory tract infection) and meningitis/encephalitis. This subject has committed a DEO (failed to include both clinically important diagnoses in his diagnostic workup).

**Figure 2:**
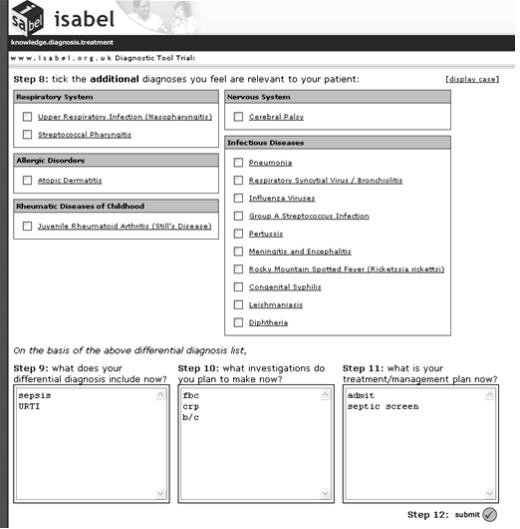
**Screenshot of ISABEL simulated study procedure – step 2**. This figure shows how the same subject was provided the results of ISABEL's search in one click, and how he was provided the opportunity to modify the clinical decisions made in step 1. It was not possible to go back from this step to step 1 to modify the clinical decisions made earlier. Notice that the subject has not identified meningitis/encephalitis from the ISABEL suggestions as clinically important. He has made no changes to his workup, and has committed a DEO despite system advice.

**Figure 3:**
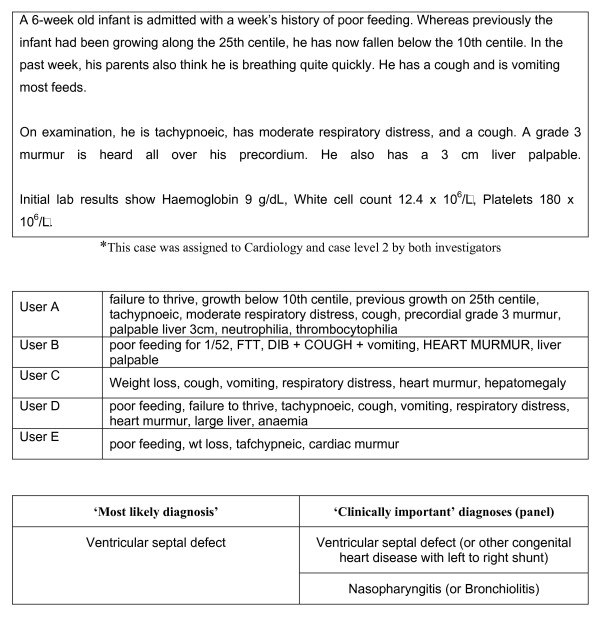
Example of one simulated case used in study*, the variability in clinical features as abstracted by five different users (verbatim), and clinically important diagnoses as judged by panel.

Subjects were allowed to complete their assessments of the simulated cases from any computer connected to the Internet at any time (i.e. they were not supervised). After logging into the trial website, subjects were presented with text from a case simulation. They assessed the case, abstracted the salient clinical features according to their own interpretation of the case, and entered them into the designated search query box in free text. Following this, they keyed in their decisions regarding diagnostic workup, test-ordering and treatment into the designated textboxes. These constituted pre-DSS clinical decisions. See figure 1 for an illustration of this step of the study procedure. On submitting this information, a list of diagnostic suggestions was instantly presented to the subject based on the abstracted clinical features. The subjects could not read the case text again at this stage, preventing them from processing the case a second time, thus avoiding 'second-look' bias. Diagnostic suggestions were different for different users since the search query was unique for each subject, depending on their understanding of the case and how they expressed it in natural language. On the basis of the diagnostic suggestions, subjects could modify their pre-DSS clinical decisions by adding or deleting items: these constituted post-DSS clinical decisions. All clinical decisions, and the time taken to complete each step, were recorded automatically. See figure 2 for an illustration of this step of the study procedure. The text from one case, and the variability associated with its interpretation during the study, is depicted in figure 3.

Each subject was presented with 12 cases such that one of the pair drawn from each sub-specialty was displayed. Cases were presented in random order (in no particular order of sub-specialty). Subjects could terminate their session at any time and return to complete the remainder of cases. If a session was terminated midway through a case, that case was presented again on the subject's return. If the website detected no activity for > 2 hours, the subject was automatically logged off, and the session was continued on their return. All subjects had 3 weeks to complete their assigned 12 cases. Since each case was used more than once, by different subjects, we termed each attempt by a subject at a case as a 'case episode'.

### Scoring metrics

We aimed to assess if the provision of key diagnostic reminders would reduce errors of omission in the simulated environment. For the purposes of this study, a subject was defined to have committed a DEO for a case episode if they failed to include *all *'clinically important diagnoses' in the diagnostic workup (rather than failing to include the 'correct diagnosis'). A diagnosis was judged 'clinically important' if an expert panel working the case independently decided that the particular diagnosis had to be included in the workup in order to ensure safe and appropriate clinical decision making, i.e. they would significantly affect patient management and/or course, and failure to do so would be construed clinically inadequate. The expert panel comprised two general pediatricians with > 3 years consultant level experience. 'Clinically important' diagnoses suggested by the panel thus included the 'most likely diagnosis/es' and other key diagnoses; they did not constitute a full differential containing all plausible diagnoses. This outcome variable was quantified by a binary measure (for each case episode, a subject either committed a DEO or not). Errors of omission were defined for tests and treatments in similar fashion.

**Figure 4:**
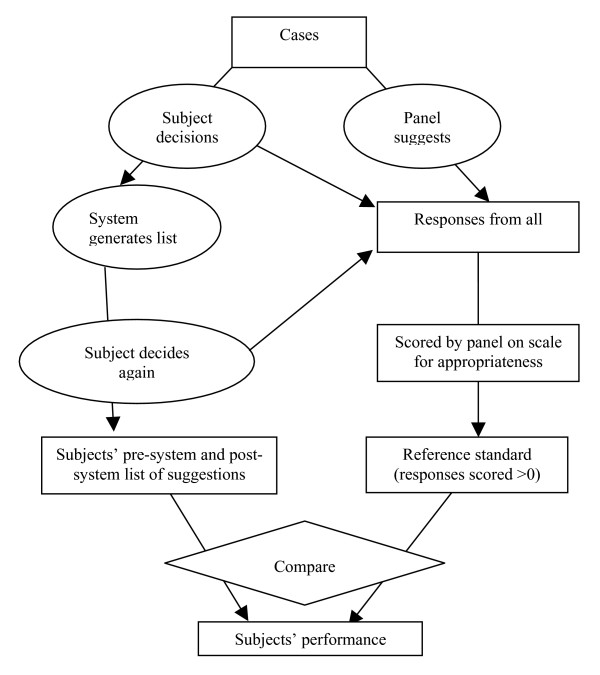
Schematic of scoring procedure.

We also sought a more sensitive assessment of changes in the quality of clinical decisions made by subjects in this study. Since an appropriate and validated instrument was essential for this purpose, a measurement study was first undertaken to develop and validate such an instrument. The measurement study, including the development and validation of a diagnostic quality score and a management plan quality score, has previously been reported in detail [52]. The scoring process, tested using a subset of cases worked on by clinicians during this study (190 case episodes), was reliable (intraclass correlation coefficient 0.79) and valid (face, construct and concurrent validity). During the scoring process, the expert panel was provided an aggregate list of decisions drawn from all subjects (pre- and post-DDSS consultation) for each case. They provided measurements of quality for each of the clinical decisions in addition to identifying 'clinically important' decisions for each case. Prior to scoring, one investigator (PR) mapped diagnoses proposed by subjects and the expert panel to the nearest equivalent diagnoses in the ISABEL database. Quality of each diagnostic decision was scored for the degree of plausibility, likelihood in the clinical setting, and its impact on further patient management. These measurements were used to derive scores for each set of subjects' clinical decisions (diagnostic workup, tests and treatments). As per the scoring system, subjects' decision plans were awarded the highest score (score range: 0 to 1) only if they were both comprehensive (contained *all *important clinical decisions), and focused (contained *only *important decisions). Scores were calculated for each subject's diagnostic, test-ordering and treatment plans both pre- and post-ISABEL consultation. Figure 4 provides a schematic diagram of the complete scoring procedure.

### Primary outcome

1. Change in the number of diagnostic errors of omission among subjects.

### Secondary outcomes

1. Mean change in subjects' diagnostic, test-ordering and treatment plan quality scores.

2. Change in the number of irrelevant diagnoses contained within the diagnostic workup.

3. Proportion of case episodes in which at least one additional 'important' diagnosis, test or treatment decision was considered by the subject *after *DSS consultation.

4. Additional time taken for DSS consultation.

### Analysis

Subjects were used as the unit of analysis for the primary outcome measure. For each subject, the total number of DEOs was counted separately for pre- and post-DSS diagnostic workup plans; only subjects who had completed all assigned cases were included in this calculation. Statistically significant changes in DEO count following DDSS consultation and interaction with grade was assessed by two-way mixed-model analysis of variance (grade being between-subjects factor and time being within-subjects factor). Mean number of DEOs was calculated for each subject grade, and DEOs were additionally analyzed according to level of case difficulty. Statistical significance was set at a p value of 0.05.

Subjects were used as the unit of analysis for the change in mean quality scores (the development of quality scores and their validation has been previously described; however, the scores have never been used as an outcome measure prior to this evaluation). In the first step, subjects' quality score (pre- and post-DSS) was calculated for each case episode. For each subject, a mean quality score across all 12 cases was computed. Only case episodes from subjects who completed all 12 assigned cases were used during this calculation. A two-way mixed model ANOVA (grade as between-subjects factor; time as within-subjects factor) was used to examine statistically significant differences in quality scores. This analysis was performed for diagnostic quality scores as well as test ordering and treatment plan scores. Data from a pilot study suggested that data from 64 subjects were needed to demonstrate a mean diagnostic quality score change of 0.03 (standard deviation 0.06, power 80%, level of significance 5%).

Using subjects as the unit of analysis, the mean count of diagnoses (and irrelevant diagnoses) included in the workup was calculated pre- and post-DSS consultation for each subject as an average across all case attempts. Only subjects who attempted all assigned cases were included in this analysis. Using this data, a mean count for diagnoses (and irrelevant diagnoses) was calculated for each subject grade. A two-way mixed model ANOVA was used to assess statistically significant differences in this outcome with respect to grade as well as occasion. Using case episodes as the unit of analysis, the proportion of case episodes in which at least one additional 'important' diagnosis, test or treatment was prompted by ISABEL was determined. The proportion of case episodes in which at least one clinically significant decision was deleted, and at least one inappropriate decision was added, after system consultation, was also computed. All data were analyzed separately for the subjects' grades.

Two further analyses were conducted to enable the interpretation of our results. First, in order to provide a direct comparison of our results with other studies, we used case episodes as the unit of analysis and examined the presence of the 'most likely diagnosis' in the diagnostic workup. The 'most likely diagnosis' was part of the set of 'clinically important' diagnoses provided by the panel, and represented the closest match to a 'correct' diagnosis in our study design. This analysis was conducted separately for each grade. Second, since it was important to verify whether any reduction of omission errors was directly prompted by ISABEL, or simply by subjects re-thinking about the assigned cases, all case episodes in which at least one additional significant diagnosis was added by the user were examined. If the diagnostic suggestion added by the user had been displayed in the DSS list of suggestions, it strongly suggested that the system, rather than subjects' re-thinking, prompted these additions.

## Results

The characteristics of subjects, cases and case episodes are summarized in table 1. Ninety seven subjects were invited for the study. Although all subjects consented and completed their training, only seventy six subjects attempted at least one case (attempters) during their allocated three weeks. This group consisted of 15 medical students, 19 SHOs, 24 registrars and 18 consultants. There was no significant difference between attempters and non-attempters with respect to grade (Chi square test, p 0.07). Only 6/76 subjects had used ISABEL regularly (at least once a week) prior to the study period (3 SHOs, 1 Registrar and 2 Consultants); all the others had registered for the service, but never used the DSS previously. A total of 751 case episodes were completed by the end of the study period. Fifty two subjects completed all assigned 12 cases to produce 624 case episodes (completers); 24 other subjects did not complete all their assigned cases (non-completers). Completers and non-completers did not differ significantly with respect to grade (Chi square test, p 0.06). However, more subjects were trained remotely in the non-completers group (Chi square test, p 0.003). The majority of non-completers had worked at least two cases (75%); slightly less than half (42%) had worked at least 6 cases. Forty-seven diagnoses were considered 'clinically important' by the panel across all 24 cases (average ~ 2 per case). For 21/24 cases, the panel had specified a single 'most likely diagnosis'; for 3 cases, two diagnoses were included in this definition.

**Table 1:** Study participants, cases and case episodes*

**Grade of subject**							
	Consultant (%)	Registrar (%)	SHO (%)	Student (%)	**Total**	**Case episodes**	**Cases**

Subjects invited to participate	27 (27.8)	33 (34)	20 (20.6)	17 (17.5)	**97**		

Subjects who attempted at least one case (attempters)	18 (23.7)	24 (31.6)	19 (25)	15 (19.7)	**76**	**751**	**24**
Subjects who attempted at least six cases	16 (25.8)	18 (29)	15 (24.2)	13 (20.9)	**62**	**715**	**24**
Subjects who completed all 12 cases (completers)	15 (28.8)	14 (26.9)	10 (19.2)	13 (25)	**52**	**624**	**24**

Regular DSS users (usage > once/week)	2	1	3	0	**6**		

* Since each case was assessed more than once, each attempt by a subject at a case was termed as a 'case episode'.

### Diagnostic errors of omission

624 case episodes generated by 52 subjects were used to examine DEO. During the pre-DSS consultation phase, all subjects performed a DEO in at least one of their cases, and 21.1% (11/52) in more than half their cases. In the pre-ISABEL consultation stage, medical students and SHOs committed the most and least number of DEO respectively (6.6 vs. 4.4); this gradient was maintained post-ISABEL consultation (5.9 vs. 4.1). Overall, 5.5 DEO were noted per subject pre-DSS consultation; this reduced to 5.0 DEO after DSS advice (p < 0.001). No significant interaction was noticed with grade (F_3, 48 _= 0.71, p = 0.55). Reduction in DEO following DSS advice within each grade is shown in table 2. Overall, more DEOs were noted for easy cases compared to difficult cases pre- and post-DSS advice (2.17 vs. 2.05 and 2.0 vs. 1.8); however, this was not true for medical students as a subgroup (2.5 vs. 2.9). Improvement following DSS advice seemed greater for difficult cases for all subjects, although this was not statistically significant. These findings are summarized in table 3.

**Table 2:** Mean count of diagnostic errors of omission (DEO) pre-ISABEL and post-ISABEL consultation

Grade of subject	DEO pre-ISABEL (SD)	DEO post-ISABEL (SD)	Reduction (SD)
Consultant	5.13 (1.3)	4.6 (1.4)	0.53 (0.7)
Registrar	5.64 (1.5)	5.14 (1.6)	0.5 (0.5)
SHO	4.4 (1.6)	4.1 (1.6)	0.3 (0.5)
Medical student	6.61 (1.3)	5.92 (1.4)	0.69 (0.7)

Mean DEO across all subjects (n = 52)*	**5.50 (1.6)**	**4.98 (1.5)**	**0.52 (0.6)**

*Total number of subjects who completed all 12 assigned cases

**Table 3:** Mean DEO count analyzed by level of case and subject grade

**Grade**	**Difficult cases**		**Easy cases**	
	
	Pre-DSS	Post-DSS	Pre-DSS	Post-DSS
Consultant	1.66	1.47	2.0	1.87
Registrar	2.21	1.93	2.14	1.92
SHO	1.3	1.2	2.0	1.8
Medical student	2.92	2.54	2.54	2.30

### Mean quality score changes

624 case episodes from 52 subjects who had completed all assigned 12 cases were used for this analysis. Table 4 summarizes mean diagnostic quality scores pre- and post-ISABEL consultation, and the change in mean quality score for diagnoses, for each grade of subject. There was a significant change in the weighted mean of the diagnostic quality score (0.044; 95% confidence interval: 0.032, 0.054; *p *< 0.001). No significant interaction between grade and occasion was demonstrated. In 9/52 subjects (17.3%), the pre-DSS score for diagnostic quality was higher than the post-DSS score, indicating that subjects had lengthened their diagnostic workup without substantially improving its quality. Overall, the mean score for test-ordering plans increased significantly from 0.345 to 0.364 (an increase of 0.019, 95% CI 0.011–0.027, t_51 _= 4.91, p < 0.001); this increase was smaller for treatment plans (0.01, 95% CI 0.007–0.012, t_51 _= 7.15, p < 0.001).

**Table 4:** Mean quality scores for diagnoses broken down by grade of subject

	Mean pre-ISABEL score	Mean post-ISABEL score	Mean score change*
Consultant	0.39	0.43	0.044
Registrar	0.40	0.44	0.038
SHO	0.45	0.48	0.032
Medical student	0.31	0.37	0.059

**Weighted average (all subjects)†**	**0.383**	**0.426**	**0.044**

* There was no significant difference between grades in terms of change in diagnosis quality score (one-way ANOVA p > 0.05)

### Number of irrelevant diagnoses

624 case episodes from 52 subjects were used for this analysis. The results are illustrated in table 5. Overall, the mean count of diagnoses included by subjects in their workup pre-DSS advice was 3.9. This increased to 5.7 post-DSS consultation (an increase of 1.8 diagnoses). The increase was largest for medical students (a mean increase of 2.6 diagnoses) and least for consultants (1.4 diagnoses). The ANOVA showed significant interaction between grade and occasion (F_3,58 _= 3.14, p = 0.034). The number of irrelevant diagnoses in the workup changed from 0.7 pre-DSS to 1.4 post-DSS advice (an increase of 0.7 irrelevant diagnoses, 95% CI 0.5–0.75). There was a significant difference in this increase across grades (most for medical students and least for consultants; 1.1 vs. 0.3 irrelevant diagnoses, F_3, 48 _= 6.33, p < 0.01). The increase in irrelevant diagnoses did not result in a corresponding increase in the number of irrelevant or deleterious tests and treatments (an increase of 0.09 tests and 0.03 treatment decisions).

**Table 5:** Increase in the average number of diagnoses and irrelevant diagnoses before and after DSS advice, broken down by grade

**Grade**	**No. of diagnoses**			**No. of irrelevant diagnoses**		
	Pre-DSS	Post-DSS	Increase	Pre-DSS	Post-DSS	Increase

Consultant	3.3	4.6	1.3	0.4	0.7	0.3
Registrar	4.3	5.9	1.6	0.8	1.3	0.5
SHO	4.4	6.1	1.7	0.6	1.4	0.8
Medical student	4.0	6.6	2.6	1.1	2.2	1.1

### Additional diagnoses, tests and treatment decisions

At least one 'clinically important' diagnosis was added by the subject to their differential diagnosis *after *ISABEL consultation in 94/751 case episodes (12.5%, 95% CI 10.1%-14.9%). 47/76 (61.8%) subjects added at least one 'clinically important' diagnosis to their diagnostic workup after consultation. Overall, 130 'clinically important' diagnoses were added after DSS advice during the experiment. In general, students were reminded to consider many more important diagnoses than consultants, although this was not statistically significant (44 vs. 26, Chi square p > 0.05); a similar gradient was seen for difficult cases, but DSS consultation seemed helpful even for easy cases. Similar proportions for tests and treatment items were smaller in magnitude (table 6). No clinically significant diagnoses were deleted after consultation. Important tests included by subjects in their pre-DSS plan were sometimes deleted from the post-DSS plan (64 individual items from 44 case episodes). A similar effect was seen for treatment steps (34 individual items from 24 case episodes). An inappropriate test was added to the post-ISABEL list in 7/751 cases.

**Table 6:** Number of case episodes in which clinically 'important' decisions were prompted by ISABEL consultation

Number of 'important' decisions prompted by ISABEL	Diagnoses	Tests	Treatment steps
**1**	69	56	42
**2**	19	12	5
**3**	2	2	2
**4**	3	0	0
**5**	1	0	0

**None**	657	637	678

No. of case episodes in which at least ONE 'significant' decision was prompted by ISABEL	94 (12.5%)	70 (9.3%)	49 (6.5%)

Total number of individual 'significant' decisions prompted by ISABEL	130	86	58

751 case episodes were used to examine the presence of the 'most likely diagnosis'. Overall, the 'most likely diagnosis/es' were included in the pre-DSS diagnostic workup by subjects in 507/751 (67.5%) case episodes. This increased to 561/751 (74.7%) case episodes after DSS advice. The improvement was fully attributable to positive consultation effects (where the 'most likely diagnosis' was absent pre-DSS but was present post-DSS); no negative consultations were observed. Diagnostic accuracy pre-ISABEL was greatest for consultants (73%) and least for medical students (57%). Medical students gained the most after DSS advice (an absolute increase of 10%). Analysis performed to elucidate whether ISABEL was responsible for the changes seen in the rate of diagnostic error indicated that all additional diagnoses were indeed present in the system's list of diagnostic suggestions.

### Time intervals

Reliable time data was available for 633/751 episodes (table 7). Median time taken for subjects to abstract clinical features and record their initial clinical decisions on the trial website was 6 min (IQR 4–10 min); median time taken to examine ISABEL's suggestions and make changes to clinical decisions was 1 min (IQR 30 sec-2 min). Time taken for ISABEL to display its suggestions was less than 2 sec on all occasions.

**Table 7:** Time taken to process case simulations broken down by grade of subject

	**Median time pre-ISABEL**	**Median time post-ISABEL**
Consultant	5 min 5 sec	42 sec
Registrar	5 min 45 sec	57 sec
SHO	5 min 54 sec	53 sec
Medical student	8 min 36 sec	3 min 42 sec

Overall	6 min 2 sec (IQR: 4:03 – 9:47)	1 min (IQR: 30 sec – 2:04)

## Discussion

We have shown in this study that errors of omission occur frequently during diagnostic workup in an experimental setting, including in cases perceived as being common in routine practice. Such errors seem to occur in most subjects, irrespective of their level of experience. We have also demonstrated that it is possible to influence clinicians' diagnostic workup and reduce errors of omission using a stand-alone diagnostic reminder system. Following DSS consultation, the quality of diagnostic, test-ordering and treatment decisions made by various grades of clinicians improved for a range of cases, such that a clinically important alteration in diagnostic decision-making resulted in 12.5% of all consultations (1 in 8 episodes of system use).

In a previous study assessing the impact of ILIAD and QMR, in which only diagnostically challenging cases were used in an experimental setting, Friedman *et al *showed that the 'correct diagnosis' was prompted by DSS use in approximately 1 in 16 consultations [48]. Although we used a similar experimental design, we used a mix of easy as well as difficult cases to test the hypothesis that incomplete workup was encountered in diagnostic conundrums as well as routine clinical problems. Previous evaluations of expert systems used the presence of the 'correct' diagnosis as the main outcome. We focused on clinical safety as the key outcome, preferring to use the inclusion of all 'clinically important' diagnoses in the workup as the main variable of interest. In acute settings such as emergency rooms and primary care, where an incomplete and evolving clinical picture results in considerable diagnostic uncertainty at assessment, the ability to generate a focused and 'safe' workup is a more clinically relevant outcome, and one which accurately reflects the nature of decision making in this environment [53]. Consequently, we defined diagnostic errors of omission at assessment as the 'failure to consider all clinically important diagnoses (as judged by an expert panel working the same cases)'. This definition resulted in the 'correct' diagnosis, as well as other significant diagnoses, being included within the 'minimum' workup. Further, changes in test-ordering and treatment decisions were uniquely measured in this study as a more concrete marker of the impact of diagnostic decision support on the patient's clinical management; we were able to demonstrate an improvement in test-ordering in 1 in 10 system consultations, indicating that diagnostic DSS may strongly influence patient management, despite only offering diagnosis-related advice. Finally, the time expended during DSS consultation is an important aspect that has not been fully explored in previous studies. In our study, subjects spent a median of 6 minutes for clinical data entry (including typing in their unaided decisions), and a median of 1 minute to process the advice provided and make changes to their clinical decisions.

The research design employed in this study allowed us to confirm a number of observations previously reported, as well as to generate numerous unique ones. These findings relate to the operational consequences of providing diagnostic assistance in practice. In keeping with other DSS evaluations, different subject grades processed system advice in different ways, depending on their prior knowledge and clinical experience, leading to variable benefit. Since ISABEL merely offered diagnostic suggestions, and allowed the clinician to make the final decisions (acting as the 'learned intermediary') [54], in some cases, subjects ignored even important advice. In some other cases, they added irrelevant decisions or deleted important decisions after DSS consultation, leading to reduced net positive effect of the DDSS on decision making. For some subjects whose pre-DSS performance was high, a ceiling effect prevailed, and no further improvement could be demonstrated. These findings complement the results of our earlier system performance evaluation which solely focused on system accuracy and not on user interaction with DDSS. One of the main findings from this study was that consultants tended to generate shorter diagnostic workup lists containing the 'most likely' diagnoses, with a predilection to omit other 'important' diagnoses that might account for the patient's clinical features, resulting in a high incidence of DEO. Medical students generated long diagnostic workup lists, but missed many key diagnoses leading to a high DEO rate. Interestingly, all subject grades gained from the use of ISABEL in terms of a reduction in the number of DEO, although to varying degrees. Despite more DEOs occurring in cases considered to be routine in practice than in rare and difficult ones in the pre-DSS consultation phase, ISABEL advice seemed to mainly improve decision making for difficult cases, with a smaller effect on easy cases. The impact of DSS advice showed a decreasing level of beneficial effect from diagnostic to test-ordering to treatment decisions. Finally, although the time taken to process cases without DSS advice in this study compared favorably with the Friedman evaluation of QMR and ILIAD (6 min vs. 8 min), the time taken to generate a revised workup with DSS assistance was dramatically shorter (1 min vs. 22 min).

We propose a number of explanations for our findings. There is sufficient evidence to suggest that clinicians with more clinical experience resort to established pattern-recognition techniques and the use of heuristics while making diagnostic decisions [55]. While these shortcuts enable quick decision making in practice, and work successfully on most occasions, they involve a number of cognitive biases such as 'premature closure' and 'confirmation bias' that may lead to incomplete assessment on some occasions. On the other hand, medical students may not have developed adequate pattern-recognition techniques or acquired sufficient knowledge of heuristics to make sound diagnostic decisions. It may well be that grades at an intermediate level are able to process cases in an acute setting with a greater emphasis on clinical safety. This explanation may also account for the finding that subjects failed to include 'important' diagnoses during the assessment of easy cases. Recognition that a case was unusual may trigger a departure from the use of established pattern-recognition techniques and clinical shortcuts to a more considered cognitive assessment, leading to fewer DEO in these cases. We have shown that it is possible to reduce DEOs by the use of diagnostic reminders, including in easy cases, although subjects appeared to be more willing to revise their decisions for difficult cases on the basis of ISABEL suggestions. It is also possible that some subjects ignored relevant advice because the system's explanatory capacity was inadequate and did not allow subjects to sufficiently discriminate between the suggestions offered. User variability in summarizing cases may also explain why variable benefits were derived from ISABEL usage – subjects may have obtained different results depending on how they abstracted and entered clinical features. This user variability during clinical data entry has been demonstrated even with use of a controlled vocabulary in QMR [56]. We observed marked differences between users' search terms for the same textual case; however, diagnostic suggestions did not seem to vary noticeably. This observation could be partially explained by the enormous diversity associated with various natural language disease descriptions contained within the ISABEL database, as well as by the system's use of a thesaurus that converts medical slang into recognized medical terms.

The diminishing level of impact from diagnostic to test-ordering to treatment decisions may be a result of system design – ISABEL does not explicitly state which tests and treatments to perform for each of its diagnostic suggestions. This advice is usually embedded within the textual description of the disease provided to the user. Study and system design may both account for the differences in time taken to process the cases. In previous evaluations, subjects processed cases without using the DSS in the first instance; in a subsequent step, they used the DSS to enter clinical data, record their clinical decisions, and processed system advice to generate a second diagnostic hypothesis list. In our study, subjects processed the case and recorded their own clinical decisions while using the DSS for clinical data entry. The second stage of the procedure only involved processing ISABEL advice and modifying previous clinical decisions. As such, direct comparison between the studies can be made only by the total time involved per case (30 min vs. 7 min). This difference could be explained by features in the system's design that resulted in shorter times to enter clinical data and to easily process the advice provided.

The findings from this study have implications specifically for ISABEL as well as other diagnostic DSS design, evaluation and implementation. It is well recognized that the dynamic interaction between user and DSS plays a major role in their acceptance by physicians [57]. We feel that adoption of the ISABEL system during clinical assessment in real time is possible even with current computer infrastructure, providing an opportunity for reduction in DEO. Its integration into an EMR would allow further control on the quality of the clinical input data as well as provision of active decision support with minimum extra effort. Such an ISABEL interface has currently been developed and tested with four commercial EMRs [58]; this integration also facilitates iterative use of the system during the evolution of a patient's condition, leading to increasingly specific diagnostic advice. The reminder system model aims to enable clinicians to generate 'safe' diagnostic workups in busy environments at high risk for diagnostic errors. This model has been successfully used to alter physician behavior by reducing errors of omission in preventive care [59]. It is clear from recent studies that diagnostic errors occur in the emergency room for a number of reasons. Cognitive biases, of which 'premature closure' and faulty context generation are key examples, contribute significantly [60]. Use of a reminder system may minimize the impact of some of these cognitive biases. When combined with cognitive forcing strategies during decision making, DDSS may act as 'safety nets' to reduce the incidence of omission errors in practice [61]. Reminders to perform important tests and treatment steps may also allow a greater impact on patient outcome [62]. A Web-based system model in our study allowed users from disparate parts of the country to participate in this study without need for additional infrastructure or financial resources, an implementation model that would minimize the cost associated with deployment in practice. Finally, the role of DDSS in medical education and training needs formal evaluation. In our study, medical students gained significantly from the advice provided, suggesting that use of DDSS during specific diagnostic tasks (e.g. problem-based case exercises) might be a valuable adjunct to current educational strategies. Familiarity with DDSS will also predispose to greater adoption of computerized aids during future clinical decision making.

The limitations of this study stem mainly from its experimental design. The repeated measures design raises the possibility that some of the beneficial effects seen in the study are a result of subjects 'rethinking' the case, or the consequence of a reflective process [63]. Consequently, ISABEL's effects in practice could be related to the extra time taken by users in processing cases. We believe that any such effects are likely to be minimal since subjects did not actually process the cases twice during the study – a summary of the clinical features was generated by subjects when the case was displayed for the first time, and subjects could not review the cases while processing ISABEL suggestions in the next step. Subjects also spent negligible time between their first assessment of the cases and processing the diagnostic suggestions from the DSS. The repeated measures design provided the power to detect differences between users with minimal resources; a randomized design using study and control groups of subjects would have necessitated the involvement of over 200 subjects. The cases used in our study contained only basic clinical data gained at the time of acute assessment, and may have proved too concise or easy to process. However, this seems unlikely since subjects only took an average of 8 min to process even diagnostic conundrums prior to DSS use when 'expert systems' were tested. Our cases pertained to emergency assessments, making it difficult to generalize the results to other ambulatory settings. The ability to extract clinical features from textual cases may not accurately simulate a real patient encounter where missed data or 'red herrings' are quite common. The inherent complexity involved in patient assessment and summarizing clinical findings in words may lead to poorer performance of the ISABEL system in real life, since its diagnostic output depends on the quality of user input. As a corollary, some of our encouraging results may be explained by our choice of subjects: a few were already familiar with summarizing clinical features into the DSS. Subjects were not supervised during their case exercises since they may have performed differently under scrutiny, raising the prospect of a Hawthorne effect [64]. The use of a structured website to explicitly record clinical decisions may have invoked the check-list effect, as illustrated in the Leeds abdominal pain system study [65]. The check list effect might also be invoked during the process of summarizing clinical features for ISABEL input; this may have worked in conjunction with 'rethinking' to promote better decision making pre-ISABEL. We also measured decision making at a single point in time, making it difficult to assess the effects of iterative usage of the DSS on the same patient. Finally, our definition of diagnostic error aimed to identify inadequate diagnostic workup at initial assessment that might result in a poor patient outcome. We recognize the absence of an evidence-based link between omission errors and diagnostic adverse events in practice, although according to the Schiff model [53], it seems logical to assume that avoiding process errors will prevent actual errors at least in some instances. In the simulated setting, it was not possible to test whether inadequate diagnostic workup would directly lead to a diagnostic error and cause patient harm. Our planned clinical impact assessment in real life would help clarify many of the questions raised during this experimental study.

## Conclusion

This experimental study demonstrates that diagnostic omission errors are common during the assessment of easy as well as difficult cases. The provision of patient- and context-specific diagnostic reminders has the potential to reduce these errors across all subject grades. Our study suggests a promising role for the use of future reminder-based DSS in the reduction of diagnostic error. An impact evaluation, utilizing a naturalistic design and conducted in real life clinical practice, is underway to verify the conclusions derived from this simulation.

## Competing interests

This study was conducted when the ISABEL system was available free to users, funded by the ISABEL Medical Charity (2001–2002). Dr Ramnarayan performed this research as part of his MD thesis at Imperial College London. Dr Britto was a Trustee of the ISABEL medical charity (non-remunerative post). Ms Tomlinson was employed by the ISABEL Medical Charity as a Research Nurse.

Since June 2004, ISABEL is managed by a commercial subsidiary of the Medical Charity called ISABEL Healthcare. The system is now available only to subscribers. Dr Ramnarayan now advises ISABEL Healthcare on research activities on a part-time basis; Dr Britto is now Clinical Director of ISABEL Healthcare; Ms Tomlinson is responsible for Content Management within ISABEL Healthcare. All three hold stock options in ISABEL Healthcare. All other authors declare that they have no competing interests.

## Authors' contributions

PR conceived the study, contributed to the study design, analyzed the data and drafted the manuscript.

GR assisted with the design of the study and data analysis.

MC assisted with the study design, served as gold standard panel member, and revised the draft manuscript

VN assisted with study design, served as gold standard panel member, and helped with data analysis

MT assisted with data collection and analysis

PT assisted with study conception, study design and revised the draft manuscript

JW assisted with study conception, provided advice regarding study design, and revised the draft manuscript

JB assisted with study conception, study design and data analysis.

All authors read and approved the final manuscript.

## Pre-publication history

The pre-publication history for this paper can be accessed here:


